# Fully synthetic phosphorylated Tau181, Tau217, and Tau231 calibrators for Alzheimer’s disease diagnosis

**DOI:** 10.3389/fnagi.2023.1340706

**Published:** 2024-01-15

**Authors:** Xinyu Li, Huimei Zeng, Pradeepraj Durairaj, Weihuan Wen, Tianpeng Li, Yanru Zhao, Yang Liu, Xue Liu, Lingpeng Zhan, Lang Rao, Wen Yuan, Tengfei Guo, Weijun Shen, Hui Cai, Zhicheng Chen

**Affiliations:** ^1^School of Pharmaceutical Sciences (Shenzhen), Sun Yat-sen University, Shenzhen, China; ^2^Center for Translational Research, Shenzhen Bay Laboratory, Shenzhen, China; ^3^Institute for Cell Analysis, Shenzhen Bay Laboratory, Shenzhen, China; ^4^Institute of Biomedical Health Technology and Engineering, Shenzhen Bay Laboratory, Shenzhen, China; ^5^Institute of Neurological Diseases, Shenzhen Bay Laboratory, Shenzhen, China; ^6^Institute of Biomedical Engineering, Shenzhen Bay Laboratory, Shenzhen, China

**Keywords:** Alzheimer’s disease, Tau, calibrator, Simoa, diagnosis, immunoassay

## Abstract

**Background:**

The calibrator in immunoassay plays an essential role in diagnosing Alzheimer’s disease (AD). Presently, the most well-studied biomarkers for AD diagnosis are three phosphorylated Tau (p-Tau): p-Tau231, p-Tau217, and p-Tau181. Glycogen synthase-3beta (GSK3β)-phosphorated Tau-441 is the most commonly used calibrator for p-Tau immunoassays. However, the batch-to-batch inconsistency issue of the commonly used GSK3β-phosphorylated Tau-441 limits its clinical application.

**Methods:**

We have successfully generated and characterized 61 Tau monoclonal antibodies (mAbs) with distinct epitopes by using the hybridoma technique and employed them as capture or detection antibodies for p-Tau immunoassays. Through chemical synthesis, we synthesized calibrators, which are three peptides including capture and detection antibody epitopes, for application in immunoassays that detect p-Tau231, p-Tau217, and p-Tau181. The novel calibrators were applied to Enzyme-linked immunosorbent assay (ELISA) and Single-molecule array (Simoa) platforms to validate their applicability and establish a range of p-Tau immunoassays.

**Results:**

By employing the hybridoma technique, 49 mAbs recognizing Tau (1–22), nine mAbs targeting p-Tau231, one mAb targeting p-Tau217, and two mAbs targeting p-Tau181 were developed. Peptides, including recognition epitopes of capture and detection antibodies, were synthesized. These peptides were used as calibrators to develop 60 immunoassays on the ELISA platform, of which six highly sensitive immunoassays were selected and applied to the ultra-sensitive Simoa platform. Remarkably, the LODs were 2.5, 2.4, 31.1, 32.9, 46.9, and 52.1 pg/ml, respectively.

**Conclusion:**

Three novel p-Tau calibrators were successfully generated and validated, which solved the batch-to-batch inconsistency issue of GSK3β-phosphorylated Tau-441. The novel calibrators exhibit the potential to promote the standardization of clinical AD diagnostic calibrators. Furthermore, we established a series of highly sensitive and specific immunoassays on the Simoa platform based on novel calibrators, which moved a steady step forward in p-Tau immunoassay application for AD diagnosis.

## 1 Introduction

As stated in the Global Alzheimer’s Report (2022), possibly up to 75% of dementia patients remain undiagnosed worldwide (Gauthier et al., 2022). The development of Alzheimer’s disease (AD) detection tools is pivotal to improving the early diagnostic rate. Biomarker-based detection approaches have advanced rapidly owing to the extensive investigation of Aβ and Tau proteins and the parallel advancement of ultrasensitive detection techniques. Remarkably, Tau with phosphorylation at threonines 231 (p-Tau231), 217 (p-Tau217), and 181 (p-Tau181) in cerebrospinal fluid (CSF) and blood are regarded as potent early biomarkers with high specificity and accuracy (Janelidze et al., 2023; Lantero-Rodriguez et al., 2023).

Remarkably, phosphorylation of these sites could be achieved by several enzymes, including the JUN amino-terminal kinase (JNK), P38 mitogen-activated protein kinase (p38 MAPK), extracellular signal-regulated kinase 2 (ERK2), and GSK3β (Reynolds et al., 2000). Of which, GSK3β-induced Tau phosphorylation decreases its affinity to microtubules and leads to microtubule destabilization (Uta et al., 1996; Rankin et al., 2007; Avila et al., 2012). Many immunoassays depend on the recombinant phosphorylated Tau-441 protein generated by the reaction of GSK3β in cells as a calibrator (Karikari et al., 2021; Leuzy et al., 2021; Lantero-Rodriguez et al., 2023). However, the GSK3β-phosphorylated Tau-441 as a calibrator has been argued to have heterogeneity and inconsistency issues, including differences in phosphorylation sites and variability in kinase activity, which may have an impact on p-Tau calibrator standardization (Liu et al., 2022). The incorporation of standardized and high-quality calibrators is therefore critical for ensuring accurate and consistent results in immunoassays.

In this study, we immunized mice with different Tau fragments as antigens to produce mAbs, of which 49 mAbs recognize Tau (1–22), nine mAbs target p-Tau231, one mAb targets p-Tau217, and two mAbs target p-Tau181. We proposed a novel strategy for synthesizing peptides as calibrators by directly linking two epitopes, capture and detection antibody epitopes. We designed novel calibrators that include three phosphorylated Tau sites: Tau (1–22)-pT231, Tau (1–22)-pT217, and Tau (1–22)-pT181, respectively. Herein, we employed the double antibody sandwich ELISA (DAS-ELISA) to validate the performance and application of calibrators owing to its high specificity, wide detection range, and high sensitivity (Maghsoudlou and Shah, 2016). Overall, the novel fully synthesized calibrators not only improved the precision and stability of immunoassays but also served as potential calibrators for the diagnosis of AD.

## 2 Methods

### 2.1 Materials and reagents

Peptides including Tau (1–45), Tau (1–22), Tau (12–34), Tau (23–44), p-Tau231-KLH, p-Tau231-BSA, p-Tau217-KLH, p-Tau217-BSA, p-Tau181-KLH, and p-Tau181-BSA were synthesized by TGpeptide. Novel Peptides Tau (1–22)-Tau (224-pT231-240), Tau (1–22)-Tau (210-pT217-227), and Tau (1–22)-Tau (174-pT181-191) were synthesized as calibrators by Sangon. They are abbreviated as Tau (1–22)-pT231, Tau (1–22)-pT217, and Tau (1–22)-pT181. The sequences of these peptides are shown in Table 1. Tau-441 was procured from Sigma and GSK3β-phosphorylated Tau-441 was provided by SignalChem. Moreover, Streptavidin, Horseradish Peroxidase Conjugated from Thermo Scientific and Goat F(ab’)2 Anti-Mouse IgG (Fab’)2 (HRP) from Abcam were employed in pivotal experimental procedures. Mice strains including BALB/C and C57BL/6 were procured from Zhuhai BesTest Bio-Tech Co., Ltd., while SP2/0 cells were sourced from Shenzhen TOP Biotechnology Co., Ltd. Additionally, the Easy-Sep Mouse CD138 Pos Selection Kit and Big EasySep Magnet were purchased from STEMCELL. Reagents such as the ELISA coating buffer, ELISA stop solution, single-component TMB chromogenic solution, 20 × PBS buffer, and DMSO were obtained from Solarbio.

**Table 1:** The sequences of peptides.

Peptides	Sequences
Tau (1–45)	MAEPRQEFEVMEDHAGTYGLGDRKDQGGYTMHQDQEGDTDAGLKE
Tau (1–22)	MAEPRQEFEVMEDHAGTYGLGD
Tau (12–34)	EDHAGTYGLGDRKDQGGYTMHQD
Tau (23–44)	RKDQGGYTMHQDQEGDTDAGLK
p-Tau231-KLH	KLH-KKVAVVRpTPPKSPSSAKC
p-Tau231-BSA	BSA-KKVAVVRpTPPKSPSSAKC
p-Tau217-KLH	KLH-CSRTPSLPpTPPTREPKKVA
p-Tau217-BSA	BSA-CSRTPSLPpTPPTREPKKVA
p-Tau181-KLH	KLH-CKTPPAPKpTPPSSGEPPKS
p-Tau181-BSA	BSA-CKTPPAPKpTPPSSGEPPKS
Tau (1–22)-Tau (224-pT231-240)	MAEPRQEFEVMEDHAGTYGLGDKKVAVVRpTPPKSPSSAK
Tau (1–22)-Tau (210-pT217-227)	MAEPRQEFEVMEDHAGTYGLGDSRTPSLPpTPPTREPKKVA
Tau (1–22)-Tau (174-pT181-191)	MAEPRQEFEVMEDHAGTYGLGDKTPPAPKpTPPSSGEPPKS

### 2.2 Hybridoma technology

Following immunizations with various forms of Tau protein in BALB/C and C57BL/6 mice, we evaluated the immune response of the mice using indirect ELISA and selected those with the highest titers. Utilizing a standard protocol, we fused splenocytes with SP2/0 myeloma cells at a ratio of 3:1 using polyethylene glycol (PEG) 4,000, resulting in the successful generation of hybridomas (Groth and Scheidegger, 1980). Under incubation conditions of 37°C with 5% CO_2_, cells were cultured in HAT medium (containing hypoxanthine, aminopterin, and thymidine) for 10 days. We subsequently employed indirect ELISA to screen for hybridomas producing mAbs against various forms of Tau. Positive hybridoma cells were then sub-cloned thrice and cultured in HT medium (containing hypoxanthine and thymidine). All animal experiments were approved by the Ethics Committee of Shenzhen Bay Laboratory.

### 2.3 Indirect ELISA

Using an indirect ELISA, we assessed serum titers, epitopes, and the EC50 affinity. Initially, we diluted various Tau proteins in coating buffer, then coated them onto a 96-well microplate (100 μL/well) and incubated overnight at 4°C. After incubation, the wells were washed three times with PBST, followed by a blocking step using 2% BSA buffer (200 μL/well) at 37°C for 1.5 h. After washing, diluted mouse serum or mAb was added to each well (100 μL/well) and incubated at 37°C for 1 h. Subsequent washing was followed by the addition of diluted Goat F(ab’)2 Anti-Mouse IgG (Fab’)2 (HRP) as the secondary antibody (100 μL/well), with an incubation at 37°C for 30 min. After a further washing step, 100 μL of freshly prepared TMB substrate solution was added to each well, and the ELISA plate was allowed to react at room temperature for 8 min. The reaction was then quenched using an ELISA stop solution (100 μL/well). Finally, absorbance was measured at a wavelength of 450 nm using a multifunctional microplate reader.

### 2.4 High-performance liquid chromatography (HPLC) and liquid chromatograph mass spectrometer (LC–MS)

The HPLC analysis of the peptide segments was performed on a Shimadzu LC-20A system using a NanoChrom Chromcore TM120 C18 and a Shim-pack GIST (4.6 × 250 mm, 5 μm) column. The mobile phase is comprised of A (0.1% trifluoroacetic acid in water) and B (0.1% trifluoroacetic acid in acetonitrile). The gradient elution conditions for peptide segments were sequentially set at 0.01–20 min (21–41% B), 0.01–20 min (23–43% B), and 0.01–20 min (23–43% B), respectively. For this analysis, a 0.1 mg sample was dissolved in 0.5 mL of 10% or 20% ACN and 90% or 80% H_2_O. The injection volume for the samples was 30 μL, with a flow rate set at 1.0 mL/min. The absorbance of peptides was detected at 214 nm.

Peptide analysis using LC–MS was performed on a Shimadzu LCMS-2020. The mobile phase for the LC–MS consisted of 50% methanol and 50% water solution, with a flow rate of 0.2 mL/min. Each analysis had an injection volume of 1 μL. The data was processed using the LabSolutions software. The following mass spectrometry parameters were utilized during the analysis: Nebulizing Gas flow rate: 1.50 L/min; Drying Gas flow rate: 5.0 L/min; Interface temperature: 350°C; Desolvation line temperature: 250°C; and Heating block temperature: 300°C.

### 2.5 DAS-ELISA

In the DAS-ELISA assay, capture antibodies were first diluted to 1 μg/mL with the ELISA coating buffer and coated onto a 96-well plate (100 μL/well), followed by an overnight incubation at 4°C. After the incubation, the wells were washed three times with PBST. A blocking step was then performed using 2% BSA buffer (200 μL/well) at 37°C for 1.5 h. Novel calibrators Tau (1–22)-pT231, Tau (1–22)-pT217, and Tau (1–22)-pT181 were initiated at concentrations of either 400 ng/mL or 4,000 ng/mL, followed by 2-fold serial dilutions. These diluted solutions (100 μL/well) were added to each respective well and incubated at 37°C for 2 h. After washing, biotin-labeled detection antibodies, diluted to 1 μg/mL (100 μL/well), were added and incubated at 37°C for 1 h. Following three washes, diluted Streptavidin and horseradish peroxidase conjugated secondary antibody (100 μL/well) was added and incubated at 37°C for 30 min. After the final washing step, 100 μL of the 3,3′,5,5’-Tetramethylbenzidine (TMB) substrate solution was added to each well for color development, and the ELISA plate was left to react at room temperature for 8 min. The reaction was terminated using the ELISA stop solution (100 μL/well), and absorbance was measured at 450 nm using a multifunctional microplate reader. A satisfactory 4PL curve fitting within the range was observed in all 60 assays. The standard curve represents three replicated independent experiments, and the LOD is determined by calculating the average of 10 blank values plus 2.5 times the standard deviation (SD).

### 2.6 Surface plasmon resonance (SPR)

SPR experiments were conducted on the Biacore 8 K+ instrument from Cytiva. Antigens were chosen as the capture ligands and antibodies were used as the analytes. Using the amine coupling method, antigens Tau-441, p-Tau231-BSA, p-Tau217-BSA, and p-Tau181-BSA were immobilized onto CM5 sensor chips with carboxymethyl dextran matrix, facilitating the analysis of binding kinetics between Tau mAbs and their respective antigens. Before the assay, 1× HBS-EP buffer was freshly prepared through a tenfold dilution of 10× HBS-EP+ buffer with ultrapure water, followed by filtering and degassing. The mAb solutions were then serially diluted in 1× HBS-EP buffer, yielding concentrations of 200 nM, 100 nM, 50 nM, 25 nM, 12.5 nM, 6.25 nM, and 3.125 nM. The association phase was set to 120 s, followed by a 120 s or 300 s dissociation phase. At a constant flow rate of 30 μL/min, samples were introduced to the chip surface using an automated sample handler. The surfaces were regenerated by a 60 s injection of 10 mM pH 2.0 glycine, and with the aid of BIA 2.0.1 (Cytiva) software, the dissociation constant (KD) and other kinetic parameters were estimated from the sensorgrams. After the experiments, chips were stored in 1 × HBS-EP buffer at 4°C.

### 2.7 Simoa HD-X immunoassay

All assays based on Simoa technology were conducted using the automated Simoa HD-X analyzer from Quanterix. All custom assay components for Simoa, including wash buffer 1, wash buffer 2, sample diluent, bead diluent, SBG diluent, discs, 96-well plates, sealing oil, cuvettes and other consumables were sourced from Quanterix Corp.

Initially, capture antibodies were conjugated to homebrew carboxylated paramagnetic beads. The buffer of the capture antibody was exchanged for the bead conjugation buffer using ultrafiltration tubes, followed by a 30-min activation of the beads with freshly prepared EDC. Subsequently, these activated beads were conjugated with the capture antibody for 2 h, followed by blocking and washing steps.

For the biotinylation of the detection antibody, its buffer was swapped for the biotinylation reaction buffer using ultrafiltration tubes. The mAb was then mixed with NHS-PEG4-Biotin from Thermo Fisher at a molar ratio of 40:1 and incubated for 30 min. Excess biotin was subsequently removed by using ultrafiltration tubes. The conjugated beads with the capture antibody and the biotinylated detection antibody were stored at 4°C until further use, with all experimental steps carried out at room temperature.

During the Simoa analysis, the capture antibody, detection antibody, and SBG were diluted to specified concentrations and transferred into plastic bottles. Concurrently, recombinant calibrators were serially diluted to the desired concentrations in the diluent and pipetted into a 96-well plate. All necessary reagents were then loaded onto the Simoa HD-X analyzer, and the analysis was executed using a two-step procedure. Ultimately, signals obtained from the beads were quantified in terms of the average enzyme per bead (AEB). For data analysis, we employed GraphPad Prism 9.5.1 software, utilizing a four-parameter logistic (4PL) curve with a weighting factor of 1/y^2^ to plot the standard curves.

## 3 Results

### 3.1 Generation of mAbs and determination of epitopes

**Figure 1:**
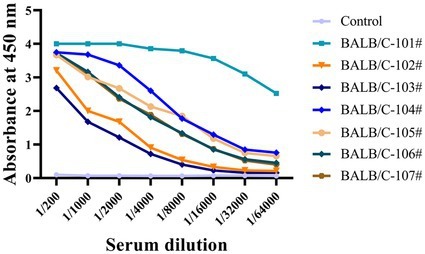
The antiserum titers of seven immunized mice were determined by indirect ELISA. Immunized BALB/C mice serum was collected, and serial dilutions were initiated at 1:200 and then added to a 96-well ELISA plate coated with recombinant Tau-441 or p-Tau231-KLH, p-Tau217-KLH, and p-Tau181-KLH proteins. Serum from pre-immunized mice was used as a negative control. The X-axis represents the antibody dilution from 1/200 to 1/64000, and the Y-axis represents the optical density at 450 nm. The results of serum titer determination demonstrated that the mice responded strongly to Tau immunization.

We used indirect ELISA to measure non-phosphorylated Tau (np-Tau) and p-Tau antibody titers after immunizing mice. Seven mice with high titers (1:32000) were chosen for fusion (Figure 1). High antibody titers appear to be characteristic of a potent immunological response in mice.

**Figure 2:**
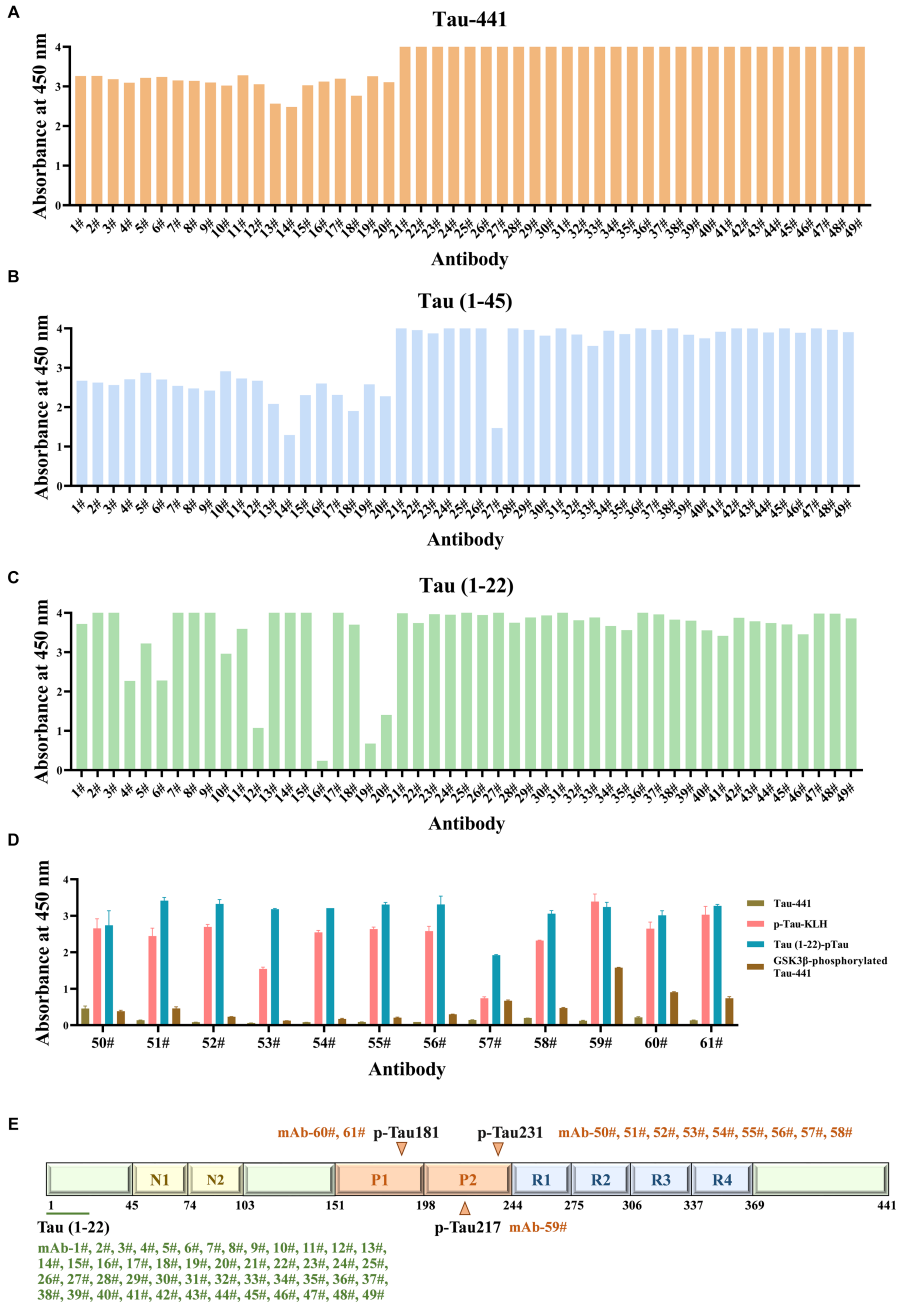
The epitopes of Tau mAbs were determined by ELISA. **(A-C)** The ability of Tau mAbs to bind to different Tau fragments was determined by indirect ELISA. 49 mAbs were bound to three Tau fragments: Tau-441, Tau (1–45), and Tau (1–22), so the binding epitopes of 49 mAbs are Tau (1–22). **(D)** The affinity of p-Tau mAbs to p-Tau-KLH, Tau (1–22)-pTau, and GSK3β-phosphorylated Tau-441 was determined. **(E)** Schematic illustration of the Tau protein sequences containing epitopes of mAbs. A total of 49 mAbs bound to Tau (1–22), nine mAbs bound to p-Tau231, one mAb bound to p-Tau217, and two mAbs bound to p-Tau181.

The formation of hybridomas in the cell culture wells was observed after fusing B lymphocytes with SP2/0 myeloma cells. The positive hybridoma clones were then identified using an indirect ELISA. After three subcloning procedures, all derived cell lines stably secreted mAbs. Following mAb production and purification, the epitopes of mAbs were investigated. Interestingly, there were 49 mAbs, mAb-1 ~ 49, that bound to np-Tau and 12 mAbs, mAb-50 ~ 61, that bound to p-Tau. As shown in Figures 2A,B, all 49 np-Tau mAbs are bound to Tau-441 and Tau (1–45). Further epitope refinement of three peptide segments: Tau (1–22), Tau (12–34), and Tau (23–44) revealed that all 49 np-Tau mAbs specifically targeted the N-terminal epitope 1–22 on human Tau protein, with amino acid numbering consistent with that full-length Tau 1–441 (Uniprot ID P10636-8) (Figure 2C, Supplementary Figure S1). The sequences of peptides are depicted in Table 1. Furthermore, epitope analysis of the 12 p-Tau mAbs revealed that nine of them (mAb-50 ~ 58) bound to p-Tau231, two of them (mAb-60 ~ 61) bound to p-Tau181, and mAb-59 bound to p-Tau217 (Figure 2D). Supplementary Table S1 shows the binding ability of the 12 p-Tau mAbs with diverse epitopes, and Figure 2E summarizes the binding epitopes of all mAbs. These mAbs with different epitopes exhibited the potential for Tau immunoassay development.

### 3.2 Synthesis and purity determination of calibrators for p-Tau231, p-Tau217, and p-Tau181 immunoassays

**Figure 3:**
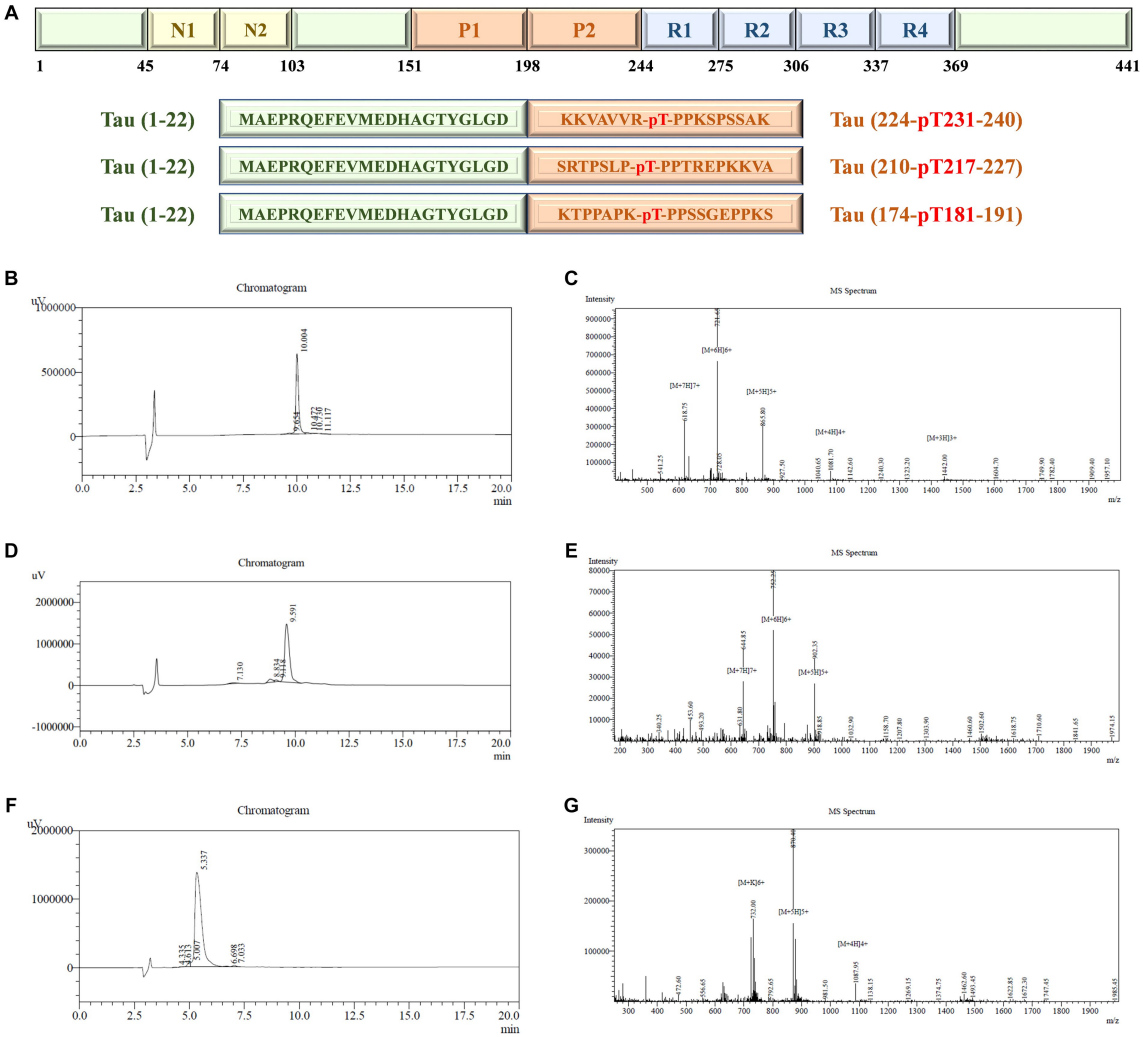
Synthesis and purity determination of calibrators. **(A)** Schematic illustration of complete chemical synthesis of peptides: Tau (1–22)-pT231, Tau (1–22)-pT217, and Tau (1–22)-pT181. **(B,C)** HPLC and LC–MS chromatograms of Tau (1–22)-pT231 peptide. **(D,E)** HPLC and LC–MS chromatograms of Tau (1–22)-pT217 peptide. **(F,G)** HPLC and LC–MS chromatograms of Tau (1–22)-pT181 peptide. HPLC, High-performance liquid chromatography; LC–MS, Liquid Chromatography with Mass Spectrometry.

The amino acid sequence of the Tau-441 protein and synthesized Tau (1–22)-pT231, Tau (1–22)-pT217, and Tau (1–22)-pT181 peptides are depicted in Figure 3A. We used chemical synthesis to generate peptides with capture and detection antibody epitopes. The purity and homogeneity of the peptides were evaluated by HPLC. The chromatogram data showed that distinct, symmetrical, and distinguishable peaks were observed. The retention times of the major peaks representing the targeted peptides were 10.0, 9.6, and 5.3 min, respectively. Based on peak area analysis, the estimated purity for these three peptides was 95.8, 91.9, and 96.1%, respectively. The absence of substantial secondary peaks in the chromatogram highlighted the high degree of purity of the peptides (Figures 3B,D,F). Further analysis using LC–MS confirmed the molecular properties of peptides that are closely aligned with the predicted amino acid sequences (Figures 3C,E,G). The combined analysis using HPLC and LC–MS confirmed the high purity and integral molecular structure of the synthesized peptides. Based on two validation methods, we subsequently applied these three synthesized peptides to develop p-Tau231, p-Tau217, and p-Tau181 immunoassays.

### 3.3 Tau mAbs showed higher affinity for our synthetic peptides than GSK3β-phosphorylated Tau-441

**Figure 4:**
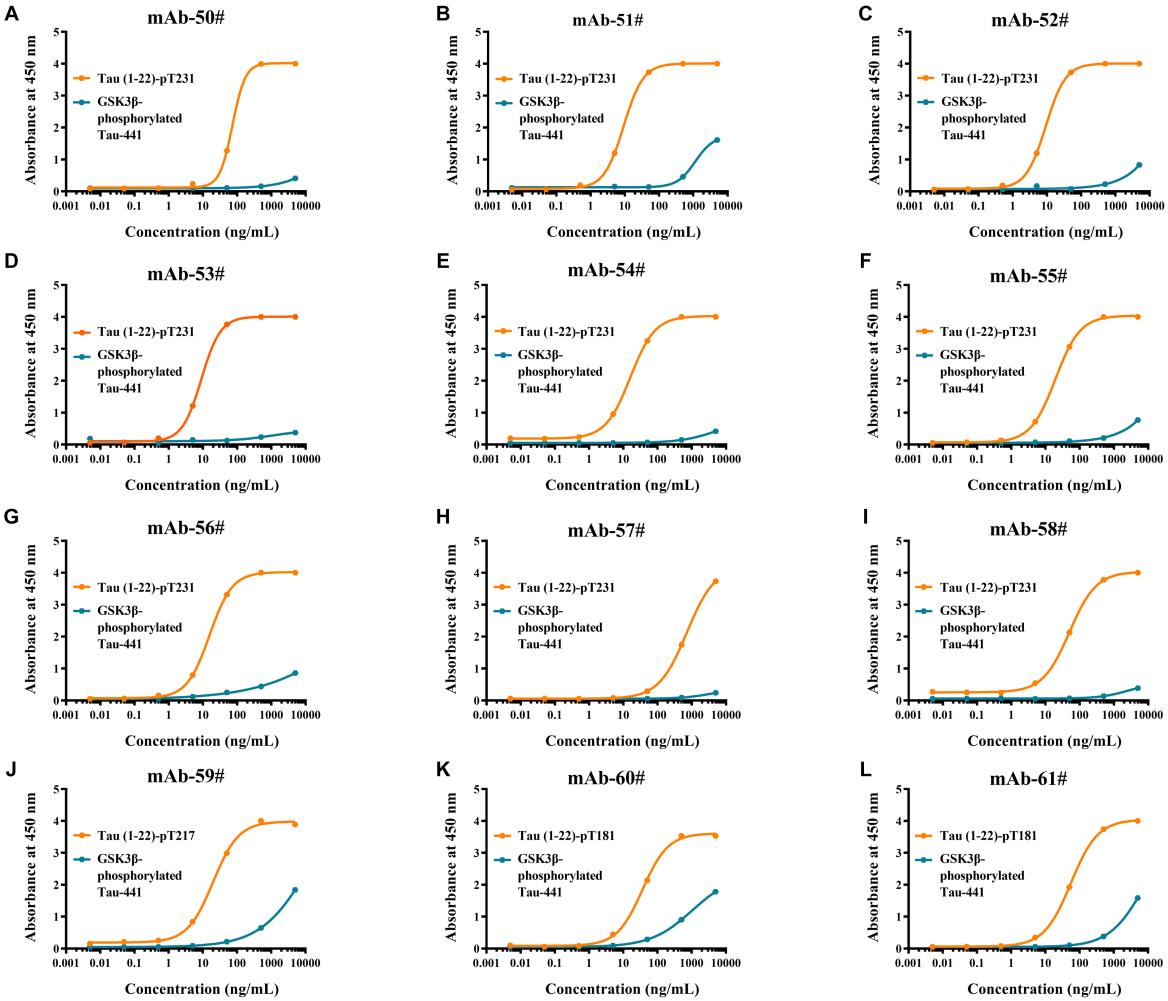
Comparative analysis of antibodies binding affinity to Tau proteins was determined by ELISA. **(A-L)** Tau (1–22)-pT231, Tau (1–22)-pT217, Tau (1–22)-pT181, and GSK3β-phosphorylated Tau-441 were coated in 96-well ELISA plates, and mAb concentrations ranged from 0.005 ng/mL to 5,000 ng/mL at a fixed antigen concentration of 1 μg/mL. Absorbance was measured from triplicate wells; error bars equal ± one standard deviation (SD).

To evaluate the synthetic Tau peptide, the binding affinities of 12 p-Tau mAbs to GSK3β-phosphorylated Tau-441 and the novel peptides Tau (1–22)-pT231, Tau (1–22)-pT217, and Tau (1–22)-pT181 were determined. Our findings showed that when the mAbs concentration is 100 ng/mL, it has a strong binding with our synthetic peptides, but GSK3β-phosphorylated Tau-441 has almost no binding (Figures 4A–L). The EC50 of mAbs binding to different p-Tau proteins is shown in Supplementary Table S2. These findings demonstrate the fact that the novel p-Tau peptides interact with the mAbs with a higher affinity and stability than the GSK3β-phosphorylated Tau-441. This increased affinity is essential for the development of ultra-sensitive DAS-ELISA.

### 3.4 Selection of 60 antibody pairs by ELISA based on synthetic peptides as calibrators

**Figure 5:**
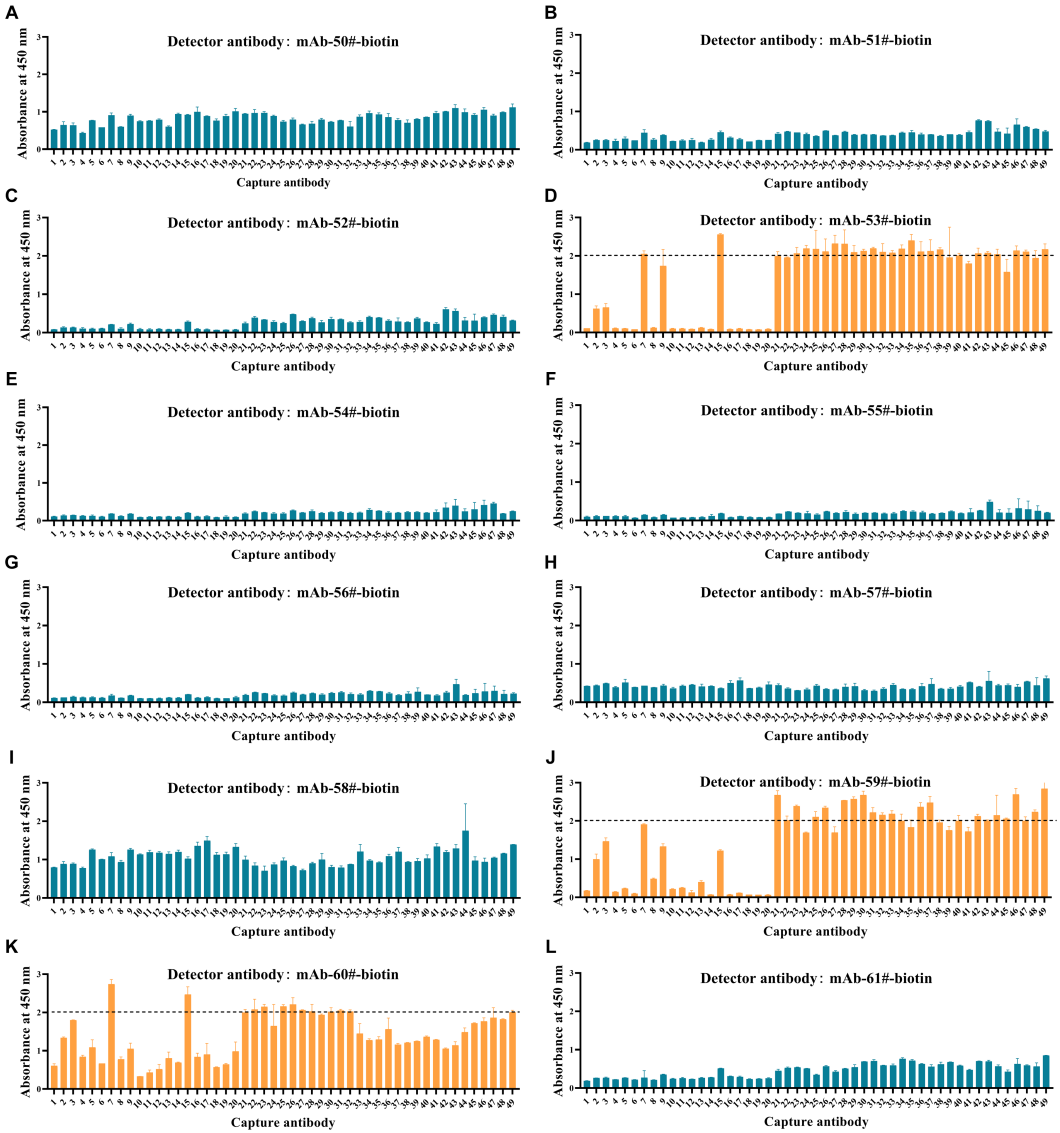
Development of p-Tau231, p-Tau217, and p-Tau181 sandwich ELISA on the ELISA platform. **(A-I)** Nine mAbs (mAb-50#, 51#, 52#, 53#, 54#, 55#, 56#, 57#, 58#) that bind to p-Tau231 are used as detection antibodies, and 49 mAbs that bind to Tau (1–22) are used as capture antibodies. The calibrator was Tau (1–22)-pT231 at a concentration of 25 ng/mL. The concentrations of the capture and detection antibodies were set at 1 μg/mL. **(J)** One mAb (mAb-59#) bound to p-Tau217 was used as the detection antibody, and 49 mAbs bound to Tau (1–22) were used as the capture antibodies. The calibrator was Tau (1–22)-pT217, at a concentration of 1 μg/mL. The concentrations of the capture and detection antibodies were set at 1 μg/mL. **(K-L)** Two mAbs (mAb-60# and mAb-61#) bound to p-Tau181 were used as detection antibodies, and 49 mAbs bound to Tau (1–22) were used as capture antibodies. The calibrator was Tau (1–22)-pT181 at a concentration of 25 ng/mL. The concentrations of the capture and detection antibodies were set at 1 μg/mL. Absorbance was measured from triplicate wells; error bars equal ± one standard deviation (SD).

To investigate the applicability of the Tau peptides as calibrators, we established immunoassays for p-Tau231, p-Tau217, and p-Tau181 using synthesized calibrators Tau (1–22)-pT231, Tau (1–22)-pT217, and Tau (1–22)-pT181. All possible combinations (a total of 588 pairs) by employing 49 np-Tau mAbs specific to Tau (1–22) as capture antibodies and 12 p-Tau mAbs as detection antibodies were investigated. The OD450 values of the 588 assays at a single concentration point are shown in Figure 5 and Supplementary Table S3. 60 assays with OD450 values greater than 2.0 from 588 assays were chosen for further concentration gradient pairing analysis. Especially in the detection antibodies of these 60 assays, all are mAb-53#, mAb-59#, and mAb-60#.

### 3.5 Development of 60 immunoassays with Tau synthetic peptides on the ELISA platform

**Figure 6:**
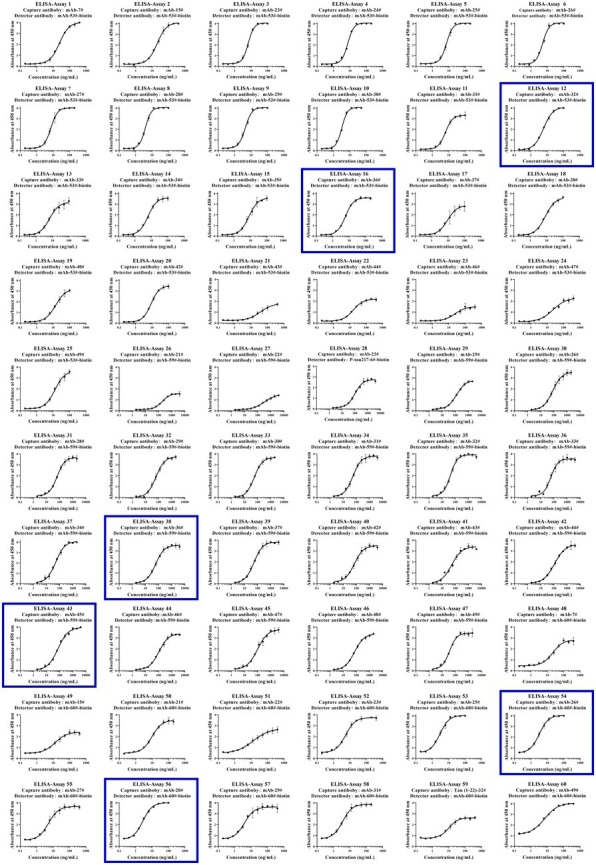
Establishment of p-Tau231, p-Tau217, and p-Tau181 immunoassays on the ELISA platform. Tau mAb-53#, mAb-59#, and mAb-60# as detection antibodies, and 49 mAbs bound to Tau (1–22) as capture antibodies. The concentration ranges of calibrators for the pT231 and pT181 immunoassays were 0.20 ~ 400 ng/mL, and the pT217 immunoassays were 2.0 ~ 4,000 ng/mL, respectively. The curve represents the 4PL curve fitting.

To further examine immunoassay sensitivity and linearity, we prepared a series of concentration gradients of the novel Tau (1–22)-pT231, Tau (1–22)-pT217, and Tau (1–22)-pT181 calibrators to establish 60 corresponding standard curves (Figure 6). For each phosphorylation site, two immunoassays were selected after evaluating the crucial selection criteria (Tables 2–4). The LODs of the six immunoassays were 0.47, 0.40, 2.94, 1.64, 0.41, and 0.37 ng/mL. These combinations are selected to be the most appropriate candidates for developing ultra-sensitivity Simoa immunoassays.

**Table 2:** The characteristics of 25 p-Tau231 immunoassays.

Assays	LOD (ng/mL)	LLOQ (ng/mL)	Background	R^2^
Assay 1	3.15	1.62	0.19	**0.9972**
Assay 2	1.79	1.37	0.20	0.9939
Assay 3	**0.12**	1.09	0.17	**0.9978**
Assay 4	2.41	1.11	0.24	0.9968
Assay 5	1.58	1.07	0.19	0.9963
Assay 6	0.53	0.69	0.18	0.9939
Assay 7	1.94	0.95	0.22	0.9949
Assay 8	0.72	0.57	0.17	0.9958
Assay 9	0.69	0.60	0.17	0.9959
Assay 10	0.97	0.50	0.20	0.9954
Assay 11	**0.39**	0.63	**0.10**	0.9921
**Assay 12**	**0.47**	**0.15**	**0.11**	**0.9978**
Assay 13	0.51	**0.30**	**0.11**	0.9776
Assay 14	0.50	**0.35**	**0.12**	0.9911
Assay 15	**0.43**	**0.43**	**0.10**	0.9874
**Assay 16**	**0.40**	**0.39**	**0.11**	**0.9971**
Assay 17	0.67	0.52	**0.11**	0.9651
Assay 18	0.65	**0.25**	**0.12**	0.9950
Assay 19	1.04	0.57	**0.12**	0.9866
Assay 20	0.95	0.55	**0.11**	0.9959
Assay 21	4.27	4.35	0.23	0.9588
Assay 22	1.73	1.50	0.19	0.9907
Assay 23	3.86	3.29	0.18	0.9428
Assay 24	1.17	0.82	0.16	0.9781
Assay 25	0.76	0.84	0.18	0.9876

There are four screening criteria: LOD < 0.5 ng/mL; LLOQ<0.5 ng/mL; Background<0.15; R2>0.997. Bold font represents the features that meet the standards. Assay 12 and Assay 16 meet all criteria.

LOD, Limit of detection; LLOQ, Lower limit of quantification; CV, Coefficient of variation.

**Table 3:** The characteristics of 22 p-Tau217 immunoassays.

Assays	LOD (ng/mL)	LLOQ (ng/mL)	Background	R^2^
Assay 26	3.80	16.70	**0.09**	0.9887
Assay 27	14.84	12.50	**0.08**	0.9876
Assay 28	**0.60**	4.97	**0.08**	0.9862
Assay 29	7.56	5.87	**0.10**	**0.9955**
Assay 30	**2.26**	**1.61**	**0.09**	0.9815
Assay 31	**1.02**	3.76	**0.09**	**0.9895**
Assay 32	5.04	**0.18**	**0.09**	**0.9900**
Assay 33	5.03	**0.69**	**0.08**	**0.9943**
Assay 34	6.05	**1.85**	0.11	0.9857
Assay 35	6.48	3.04	0.12	**0.9912**
Assay 36	**2.87**	4.20	**0.09**	0.9781
Assay 37	4.81	**1.31**	0.11	**0.9924**
**Assay 38**	**2.94**	**1.76**	**0.09**	**0.9907**
**Assay 39**	**2.00**	**1.43**	**0.09**	**0.9914**
Assay 40	7.79	2.92	0.13	0.9828
Assay 41	**1.53**	**0.61**	**0.08**	0.9780
**Assay 42**	**2.33**	**0.95**	**0.09**	**0.9925**
**Assay 43**	**1.64**	**0.89**	**0.09**	**0.9941**
Assay 44	**1.90**	2.53	**0.09**	**0.9933**
Assay 45	**0.04**	2.59	0.10	0.9850
Assay 46	3.55	**1.08**	0.11	**0.9968**
Assay 47	**0.55**	2.52	0.10	0.9888

There are four screening criteria: LOD < 3.0 ng/mL; LLOQ<2.0 ng/mL; Background<0.1; R2>0.990. Bold font represents the features that meet the standards. There are four assays that meet the requirements, taking into account the repeated consistency of the data. Finally, Assay 38 and Assay 43 were screened.

**Table 4:** The characteristics of 12 p-Tau181 immunoassays.

Assays	LOD (ng/mL)	LLOQ (ng/mL)	Background	R^2^
Assay 48	**0.17**	3.60	**0.36**	0.9771
Assay 49	0.87	2.66	**0.40**	0.9714
Assay 50	**0.29**	1.42	**0.39**	0.9873
Assay 51	0.64	4.58	**0.48**	0.9684
**Assay 52**	**0.37**	**0.86**	**0.52**	**0.9908**
**Assay 53**	**0.30**	**0.44**	**0.56**	**0.9916**
**Assay 54**	**0.41**	**0.46**	**0.56**	**0.9941**
Assay 55	**0.13**	**0.86**	**0.45**	0.9808
**Assay 56**	**0.37**	**0.47**	**0.59**	**0.9970**
Assay 57	**0.24**	**0.79**	**0.55**	0.9711
Assay 58	**0.43**	**0.71**	0.66	0.9846
Assay 59	1.49	3.31	0.67	0.9817
Assay 60	0.77	59.60	1.11	**0.9965**

There are four screening criteria: LOD < 0.5 ng/mL; LLOQ<1.0 ng/mL; Background<0.6; R2>0.990. Bold represents the features that meet the standards. There are four assays that meet the requirements, taking into account the repeated consistency of the data. Finally, Assay 54 and Assay 56 were screened.

### 3.6 Application of synthetic calibrators for Simoa immunoassays

**Figure 7:**
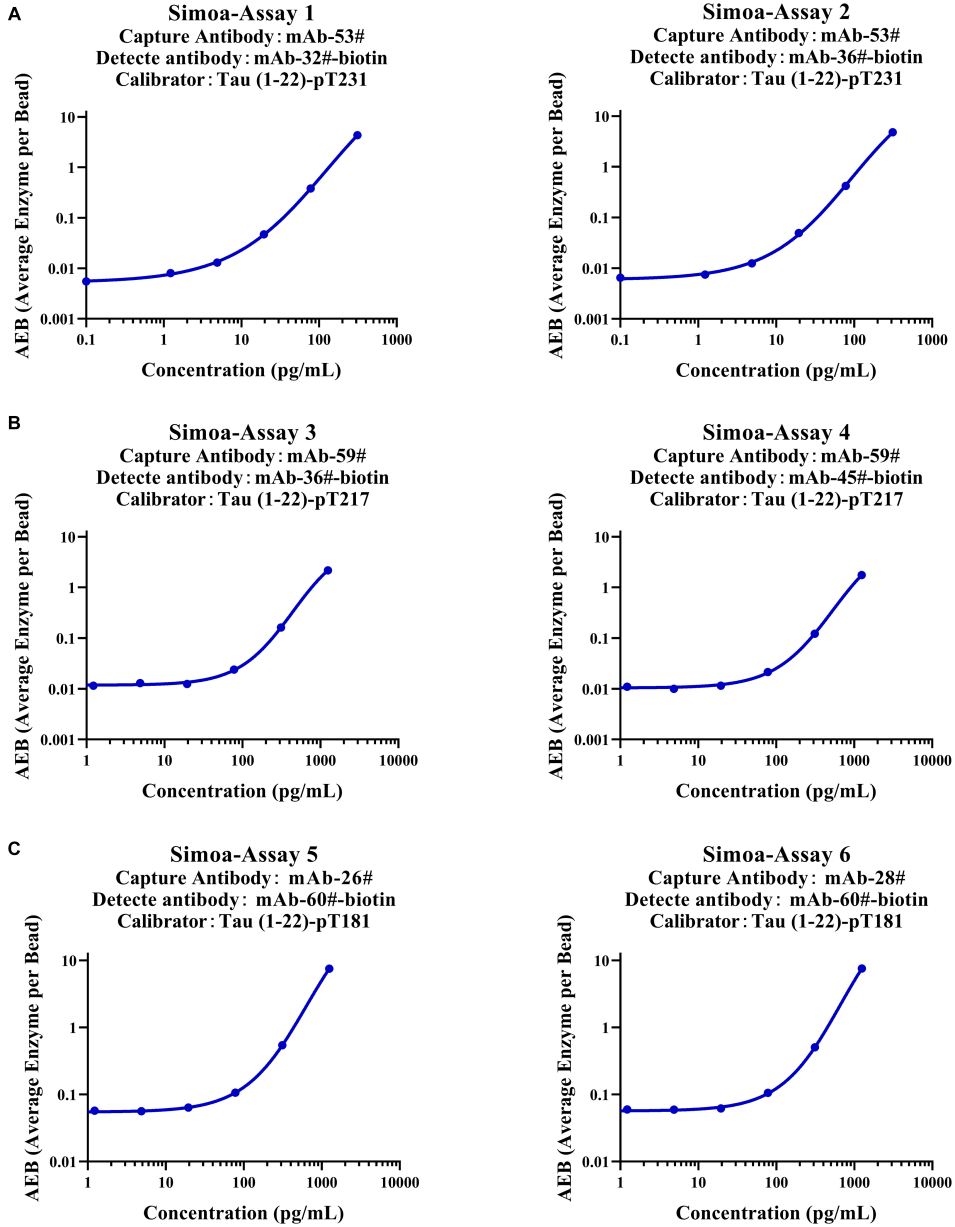
Establishment of p-Tau231, p-Tau217, and p-Tau181 immunoassays on the Simoa platform. **(A)** The capture antibody was mAb-53#, and the detection antibody was mAb-32# or mAb-36#. **(B)** The capture antibody was mAb-59#, and the detection antibody was mAb-36# or mAb-45#. **(C)** The capture antibody was mAb-26# or mAb-28#, and the detection antibody was mAb-60#. The fitting model for the six standard curves was a weighted four-parameter logistics (1/Y^2^).

To verify whether our calibrators can be applied to ultra-sensitive platforms, we developed six immunoassays on the Simoa platform, which are Simoa-Assay 1–6: capture antibody mAb-53# and detection antibody mAb-32#; capture antibody mAb-53# and detection antibody mAb-36#; capture antibody mAb-59# and detection antibody mAb-36#; capture antibody mAb-59# and detection antibody mAb-45#; capture antibody mAb-26# and detection antibody mAb-60#; capture antibody mAb-28# and detection antibody mAb-60#, respectively (Figure 7). Exceptionally, the assays achieved LLOQs of 2.5, 2.4, 31.1, 32.9, 46.9, and 52.1 pg/ml, respectively. Our findings demonstrate that the Tau (1–22)-pT231, Tau (1–22)-pT217, and Tau (1–22)-pT181 calibrators can be used to achieve sensitive quantification of p-Tau levels.

## 4 Discussion

Clinical application of AD biomarkers is often challenging and problematic as there are exists no universal cutoff values for diagnostic decision-making (Schimmel et al., 2010; Janelidze et al., 2023). Standardization of calibrators is critical for immunoassays to generate results with high accuracy and uniformity (Schimmel et al., 2010; Boulo et al., 2020; Mielke et al., 2021). In addition, the GSK3β-phosphorylated Tau-441, owing to its biological origin, could potentially be impacted by the inherent variability characteristic leading to batch-to-batch inconsistency issue (Liu et al., 2022). In previous studies, the peptide sequence “GGGSGGGSGGGS” or PEG had been successfully employed as linkers to conjugate capture and detection antibody epitopes (Hanes et al., 2020; Triana-Baltzer et al., 2020). However, the incorporation of polymers or peptides between these epitopes may result in non-specific binding or interfere with the antigen–antibody interaction. Besides, the aforementioned approach further complicates the preparation procedure. To overcome these limitations, we synthesized novel peptides that include capture and detection antibody epitopes as calibrators: Tau (1–22)-pT231, Tau (1–22)-pT217, and Tau (1–22)-pT181, instead of using traditional linkers.

Our synthesized peptides have various advantages compared with GSK3β-phosphorylated Tau-441. Primarily, these novel peptides exhibit outstanding specificity and sensitivity with minimal background signal, ensuring a higher affinity for the Tau mAbs. Interestingly, the novel peptides including capture and detection antibody epitopes without linkers, exhibited efficient concentration-dependent binding even at extremely low concentrations, whereas the GSK3β-phosphorylated Tau-441 demonstrated relatively weaker binding even at higher doses. Furthermore, chemical synthetic peptide segments ensure that products are highly consistent between batches and more cost-effective than biological processes. Overall, our novel synthetic peptides outperform GSK3β-phosphorylated Tau-441 in terms of binding with p-Tau mAbs.

For hybridoma screening, we screened all antibodies that bind to 1–22 amino acids of Tau protein, as well as antibodies that only bind to a single phosphorylated amino acid (such as site 231/217/181), we also observed a higher occurrence of antibodies binding to non-phosphorylated Tau proteins compared to phosphorylated ones. It is of the utmost importance to have mAbs with high affinity. Following that, the dissociation constant (KD) of these mAbs was determined that using the label-free biosensor technique SPR. Previous studies have demonstrated the pairs that included capture antibodies with low koff (apparent dissociation rate constants) and detection antibodies with high kon (apparent association rate constants) performed best in a DAS-ELISA format. The conditions are favorable for the capture antibody to withstand multiple incubation and washing procedures and for the detection antibody to rapidly bind to the complex within a limited time frame (Choi et al., 2008; Markwalter et al., 2017). Nevertheless, our experimental findings (as shown in Supplementary Figure S2 and Supplementary Table S4) contradicted this hypothesis. For instance, among all the nine p-Tau231 mAbs examined, mAb-53# mAb displayed a significantly low kon value as a detection antibody. We suggest that these differences are due to distinct mAb characteristics like epitopes, which make it difficult to predict mAb screening outcomes merely based on SPR kinetic parameters.

Two immunoassays with the highest affinity and specificity for each phosphorylation site were meticulously selected after an initial screening of 588 pairs of single concentration points, followed by a second screening of 60 pairs of concentration gradients. The sensitivity range of this technique was 0.3 to 3.0 ng/mL, which may limit its clinical application. Then the Simoa was employed. The lower limit of quantification (LLOQ) of Simoa ranged from 2.4 to 52.1 pg/ml, opening up possibilities for clinical application by detecting p-Tau in human CSF or plasma.

This study solved the problems of batch inconsistency and time-consuming preparation of the calibrator GSK3β-phosphorylated Tau-441 for p-Tau immunoassay. Our calibrators are chemically synthetic without a linker, with the advantages of easy synthesis, high cost-efficiency, consistency between batches, and high specificity. We will evaluate the application value of these six immunoassays in clinical samples. We also intend to synthesize calibrators containing other N-terminal Tau sequences, such as Tau (23–45), to explore their application value.

## 5 Conclusion

In conclusion, the development of novel calibrators, Tau (1–22)-pT231, Tau (1–22)-pT217, and Tau (1–22)-pT181, promoted the standardization of calibrators for p-Tau immunoassay. This breakthrough lays the important groundwork for future clinical AD early diagnosis.

## Data availability statement

The original contributions presented in the study are publicly available. This data can be found at: ProteomeXchange, PXD047248.

## Ethics statement

The animal study was approved by Ethics Committee of Shenzhen Bay Laboratory. The study was conducted in accordance with the local legislation and institutional requirements.

## Author contributions

XL: Writing – original draft, Writing – review & editing, Formal analysis, Investigation, Data curation. HZ: Writing – original draft, Writing – review & editing, Investigation, Project administration. PD: Writing – original draft, Writing – review & editing. WW: Writing – original draft, Writing – review & editing. TL: Writing – review & editing, Visualization. YZ: Writing – review & editing, Visualization. YL: Writing – review & editing, Visualization. XL: Writing – review & editing, Visualization. LZ: Writing – review & editing, Validation. LR: Writing – review & editing, Investigation. WY: Writing – review & editing, Investigation. TG: Writing – review & editing, Investigation.WS: Writing – review & editing, Conceptualization, Supervision, Resources. HC: Writing – review & editing, Conceptualization, Supervision, Resources. ZC: Writing – review & editing, Conceptualization, Supervision, Resources.
