# Supplementary material for: Fully synthetic phosphorylated Tau181, Tau217, and Tau231 calibrators for Alzheimer’s disease diagnosis
Source: Front Aging Neurosci. 2024 Jan 15;15:1340706. doi: 10.3389/fnagi.2023.1340706 (PMC10823022; doi:10.3389/fnagi.2023.1340706)
Supplement: Supplementary file 1 [file Data_Sheet_1.docx]

Supplementary Material

# Supplementary Figures

**Supplementary Figure 1.** The binding ability of np-Tau mAbs to Tau (12-34), Tau (23-44), and control was determined by ELISA. The x-axis represents the antibody, and the y-axis represents the absorbance at 450 nm.


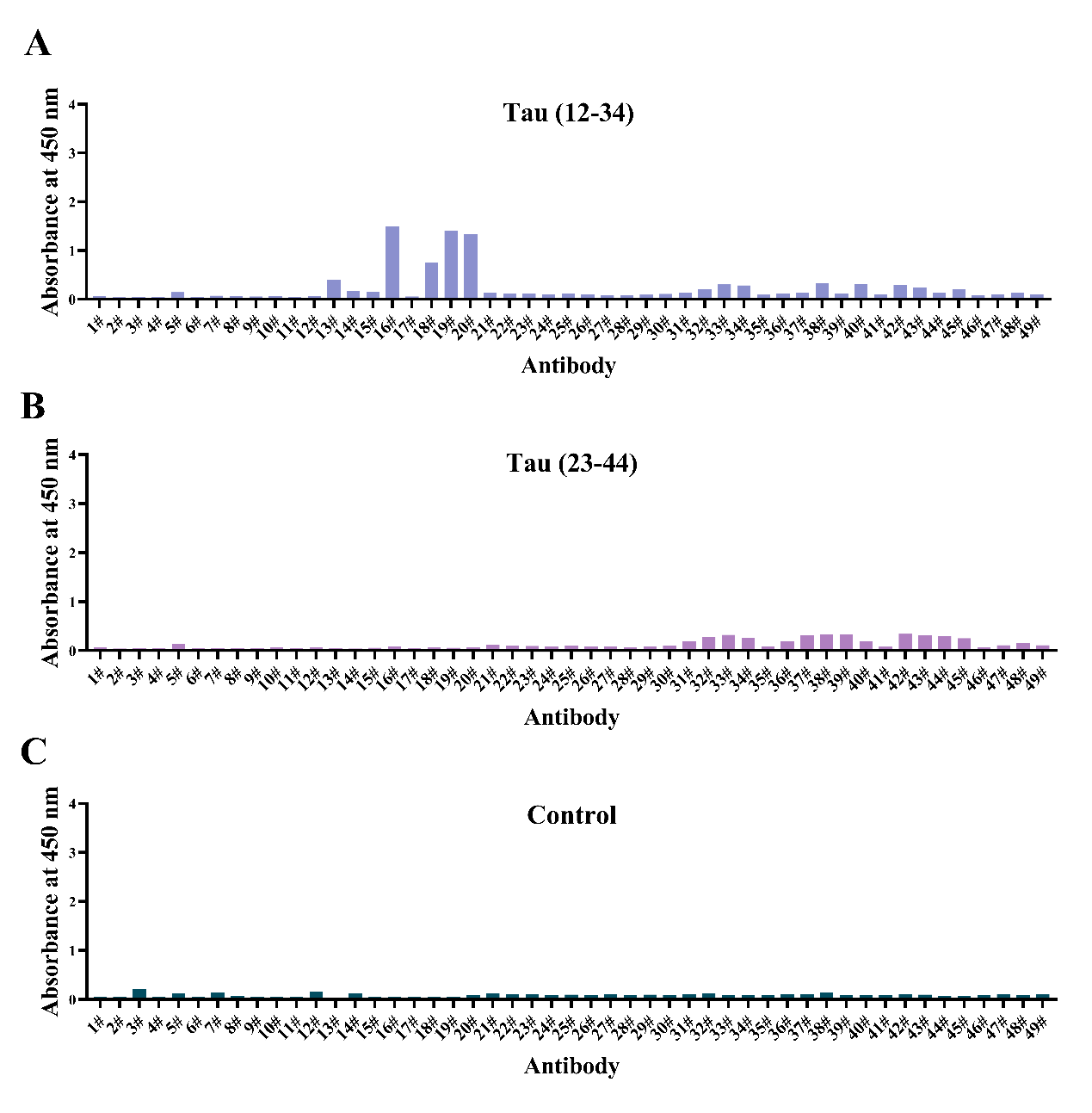


**Supplementary Figure 2.** The affinity of Tau mAbs was determined by SPR. The antigens were p-Tau231-BSA, p-Tau217-BSA, and p-Tau181-BSA. The x-axis represents times, and the y-axis represents the response value.


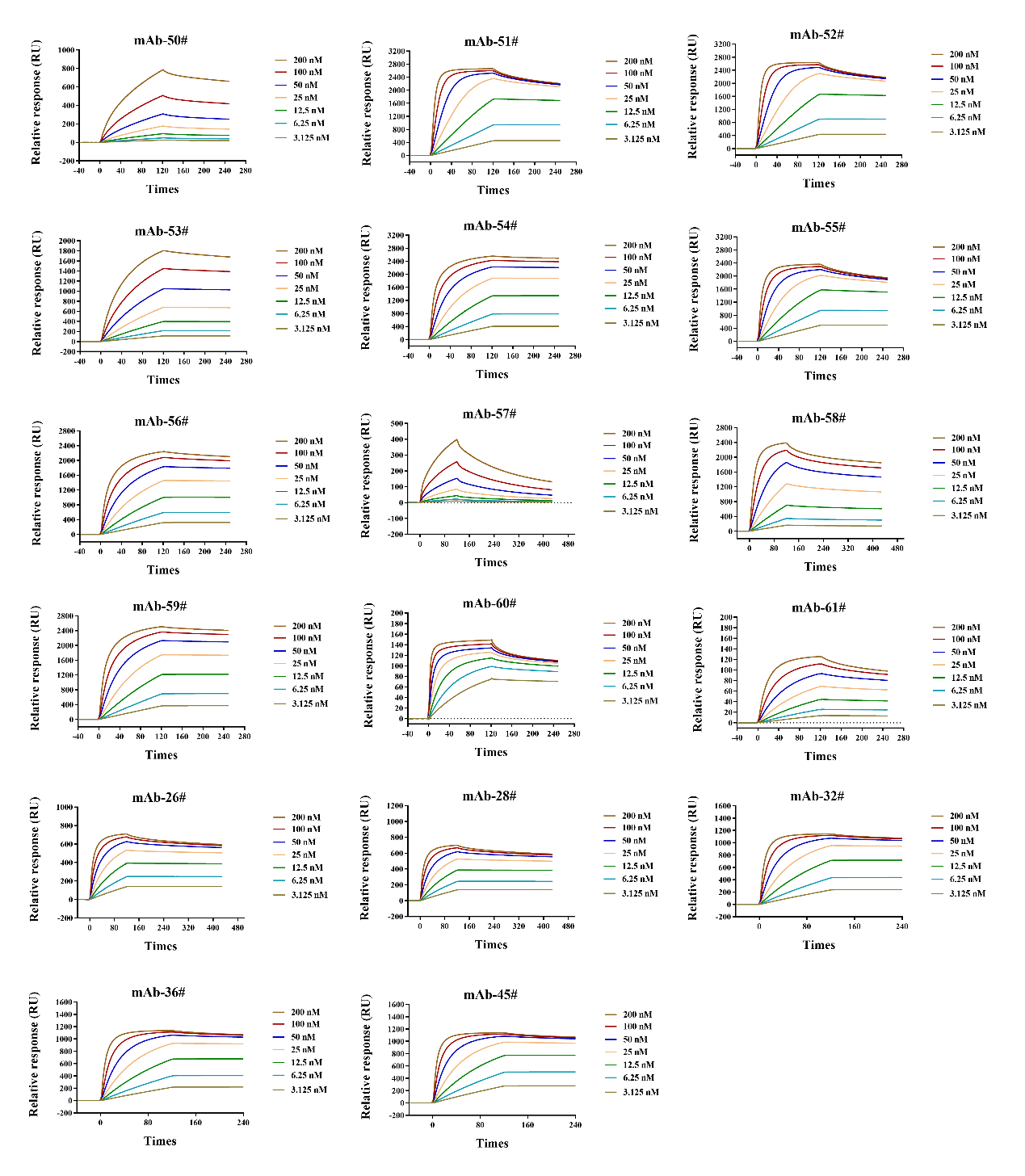


**Supplementary Figure 3.** The affinity of mAbs was determined by ELISA. The x-axis represents concentration, and the y-axis represents absorbance at 450nm.


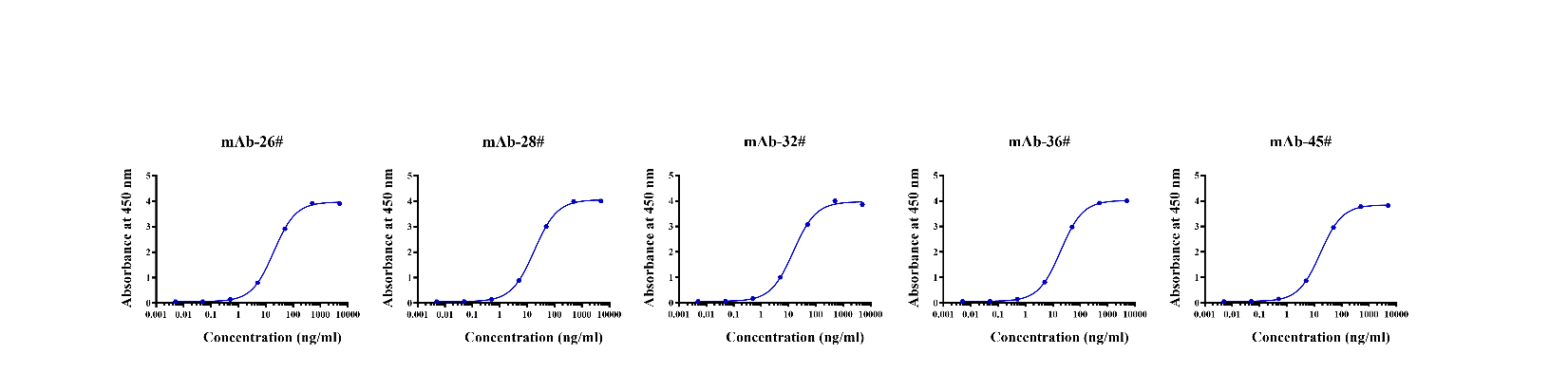


# Supplementary Tables.

**Supplementary Table 1.** The epitopes of 12 p-Tau mAbs were determined by ELISA. OD values less than 0.5 are expressed as -, between 0.5 and 1.0 are expressed as +, between 0.5 and 2.0 are expressed as ++, and between 2.0 and 3.0 are expressed as +++.

| **Antibody** | **Tau-441** | **p-Tau-KLH** | **Tau (1-22)-pTau** | **GSK3β-phosphorylated Tau-441** | **PBS** | **Epitope** |
| --- | --- | --- | --- | --- | --- | --- |
| mAb-50# | **-** | **+++** | **+++** | **-** | **-** | p-Tau231 |
| mAb-51# | **-** | **+++** | **+++** | **-** | **-** | p-Tau231 |
| mAb-52# | **-** | **+++** | **+++** | **-** | **-** | p-Tau231 |
| mAb-53# | **-** | **++** | **+++** | **-** | **-** | p-Tau231 |
| mAb-54# | **-** | **+++** | **+++** | **-** | **-** | p-Tau231 |
| mAb-55# | **-** | **+++** | **+++** | **-** | **-** | p-Tau231 |
| mAb-56# | **-** | **+++** | **+++** | **-** | **-** | p-Tau231 |
| mAb-57# | **-** | **+** | **++** | **+** | **-** | p-Tau231 |
| mAb-58# | **-** | **+++** | **+++** | **-** | **-** | p-Tau231 |
| mAb-59# | - | **+++** | **+++** | ++ | **-** | p-Tau217 |
| mAb-60# | - | **+++** | **+++** | + | **-** | p-Tau181 |
| mAb-61# | - | **+++** | **+++** | + | **-** | P-Tau181 |

**Supplementary Table 2.** The EC50 of 12 p-Tau mAbs to Tau (1-22)-pT231, Tau (1-22)-pT217, Tau (1-22)-pT181, and GSK3β-phosphorylated Tau-441 were determined by ELISA.

| **Antibody** | **EC50 to**  **Tau (1-22)-pTau (nM)** | **EC50 to**  **GSK3β-phosphorylated Tau-441 (nM)** |
| --- | --- | --- |
| mAb-50# | 0.505 | - |
| mAb-51# | 0.063 | 7.849 |
| mAb-52# | 0.063 | - |
| mAb-53# | 0.061 | 7.048 |
| mAb-54# | 0.109 | 44.658 |
| mAb-55# | 0.133 | - |
| mAb-56# | 0.107 | - |
| mAb-57# | 4.921 | 31.753 |
| mAb-58# | 0.344 | 16.959 |
| mAb-59# | 0.136 | 86.171 |
| mAb-60# | 0.250 | 7.089 |
| MAb-61# | 0.382 | 52.575 |

**Supplementary Table 3**. The ELISA results (OD450) of primary screening. Nine mAbs binding to p-Tau231, one mAb binding to p-Tau217, and two mAbs binding to p-Tau181 were used as detection antibodies, and 49 mAbs binding to Tau (1-22) were used as capture antibody. There are a total of 588 (49*12) assays. The calibrator concentration was 1 μg/ml in p-Tau217 immunoassays and 25 ng/ml in p-Tau231 and p-Tau181 immunoassays. This figure shows the OD450 nm value of each assay. The darker the color, the greater the OD450 nm value and the stronger the binding ability.

**Supplementary Table 4.** Kinetic analyses of Tau mAbs were determined by SPR.

| Antibody | | KD (M) | 1:1 binding ka (1/Ms) | Kd (m/s) |
| --- | --- | --- | --- | --- |
| **Capture Antibody** | mAb-26# | 4.41e-10 | 6.45e+5 | 2.85e-4 |
|  | mAb-28# | 4.31e-10 | 6.40e+5 | 2.76e-4 |
|  | mAb-32# | 1.35e-10 | 6.67e+5 | 9.03e-5 |
|  | mAb-36# | 1.21e-10 | 6.16e+5 | 7.43e-5 |
|  | mAb-45# | 1.27e-10 | 7.88e+5 | 1.00e-4 |
| **Detection Antibody** | mAb-50# | 2.60e-8 | 6.41e+4 | 1.66e-3 |
|  | mAb-51# | 6.87e-10 | 1.91e+6 | 1.31e-3 |
|  | mAb-52# | 7.56e-10 | 1.71e+6 | 1.29e-3 |
|  | mAb-53# | 1.22e-9 | 1.61e+5 | 1.97e-4 |
|  | mAb-54# | 6.26e-13 | 5.09e+5 | 3.18e-7 |
|  | mAb-55# | 5.55e-10 | 1.49e+6 | 8.27e-4 |
|  | mAb-56# | 2.35e-10 | 4.23e+5 | 9.97e-5 |
|  | mAb-57# | 5.87e-8 | 7.20e+4 | 4.23e-3 |
|  | mAb-58# | 2.61e-9 | 3.52e+5 | 9.17e-4 |
|  | mAb-59# | 1.88e-13 | 4.56e+5 | 2.45e-5 |
|  | mAb-60# | 7.34e-10 | 2.33e+6 | 1.71e-3 |
|  | mAb-61# | 4.08e-9 | 3.29e+5 | 1.34e-3 |
